# Agardite-(Y), Cu^2+^_6_Y(AsO_4_)_3_(OH)_6_·3H_2_O

**DOI:** 10.1107/S1600536813023477

**Published:** 2013-08-31

**Authors:** Shaunna M. Morrison, Kenneth J. Domanik, Marcus J. Origlieri, Robert T. Downs

**Affiliations:** a Department of Geosciences, University of Arizona, 1040 E. 4th Street, Tucson, Arizona 85721-0077, USA; b Lunar and Planetary Laboratory, University of Arizona, 1629 E. University Blvd., Tucson, AZ. 85721-0092, USA

## Abstract

Agardite-(Y), with a refined formula of Cu^2+^_5.70_(Y_0.69_Ca_0.31_)[(As_0.83_P_0.17_)O_4_]_3_(OH)_6_·3H_2_O [ideally Cu^2+^_6_Y(AsO_4_)_3_(OH)_6_·3H_2_O, hexa­copper(II) yttrium tris­(arsenate) hexa­hydroxide trihydrate], belongs to the mixite mineral group which is characterized by the general formula Cu^2+^_6_ *A*(*T*O_4_)_3_(OH)_6_·3H_2_O, where nine-coordinated cations in the *A*-site include rare earth elements along with Al, Ca, Pb, or Bi, and the *T*-site contains P or As. This study presents the first structure determination of agardite-(Y). It is based on the single-crystal X-ray diffraction of a natural sample from Jote West mine, Pampa Larga Mining District, Copiapo, Chile. The general structural feature of agardite-(Y) is characterized by infinite chains of edge-sharing CuO_5_ square pyramids (site symmetry 1) extending down the *c* axis, connected in the *ab* plane by edge-sharing YO_9_ polyhedra (site symmetry -6..) and corner-sharing AsO_4_ tetra­hedra (site symmetry *m*..). Hy­droxyl groups occupy each corner of the CuO_5_-square pyramids not shared by a neighboring As or Y atom. Each YO_9_ polyhedron is surrounded by three tubular channels. The walls of the channels, parallel to the *c* axis, are six-membered hexa­gonal rings comprised of CuO_5_ and AsO_4_ polyhedra in a 2:1 ratio, and contain free mol­ecules of lattice water.

## Related literature

For background to the mixite mineral group, see: Dietrich *et al.* (1969 ▶); Hess (1983 ▶); Aruga & Nakai (1985 ▶); Mereiter & Preisinger (1986 ▶); Olmi *et al.* (1988 ▶); Miletich *et al.* (1997 ▶); Kunov *et al.* (2002 ▶); Frost *et al.* (2005 ▶); Sejkora *et al.* (2005 ▶); Plášil *et al.* (2009 ▶). For research on the sorption of toxic chemicals by minerals, see: Leone *et al.* (2013 ▶). For information on mineral nomenclature, see: Hatert & Burke (2008 ▶).

## Experimental

### 

#### Crystal data

Cu_5.70_(Y_0.69_Ca_0.31_)[(As_0.83_P_0.17_)O_4_]_3_(OH)_6_·3H_2_O
*M* *_r_* = 985.85
Hexagonal, 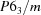

*a* = 13.5059 (5) Å
*c* = 5.8903 (2) Å
*V* = 930.50 (6) Å^3^
*Z* = 2
Mo *K*α radiation
μ = 13.13 mm^−1^
*T* = 293 K
0.10 × 0.02 × 0.02 mm

#### Data collection

Bruker APEXII CCD diffractometer
Absorption correction: multi-scan (*SADABS*; Bruker, 2004 ▶) *T*_min_ = 0.353, *T*_max_ = 0.779
20461 measured reflections
786 independent reflections
674 reflections with *I* > 2σ(*I*)
*R*_int_ = 0.048

#### Refinement

*R*[*F* ^2^ > 2σ(*F* ^2^)] = 0.032
*wR*(*F* ^2^) = 0.086
*S* = 1.14
786 reflections
60 parameters
1 restraint
H-atom parameters not refined
Δρ_max_ = 2.34 e Å^−3^
Δρ_min_ = −0.79 e Å^−3^

### 

Data collection: *APEX2* (Bruker, 2004 ▶); cell refinement: *SAINT* (Bruker, 2004 ▶); data reduction: *SAINT*; program(s) used to solve structure: *SHELXS97* (Sheldrick, 2008 ▶); program(s) used to refine structure: *SHELXL97* (Sheldrick, 2008 ▶); molecular graphics: *XtalDraw* (Downs & Hall-Wallace, 2003 ▶); software used to prepare material for publication: *publCIF* (Westrip, 2010 ▶).

## Supplementary Material

Crystal structure: contains datablock(s) I. DOI: 10.1107/S1600536813023477/wm2763sup1.cif

Structure factors: contains datablock(s) I. DOI: 10.1107/S1600536813023477/wm2763Isup2.hkl

Additional supplementary materials:  crystallographic information; 3D view; checkCIF report

## Figures and Tables

**Fig. 1.**
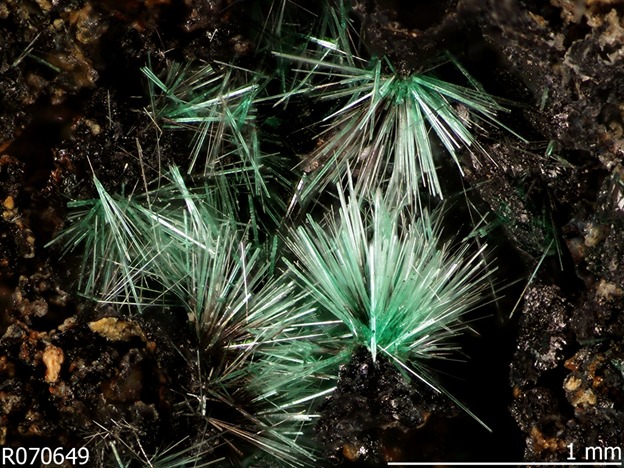
Photograph of the agardite-(Y) specimen analyzed in this study, illustrating its acicular habit.

**Fig. 2.**
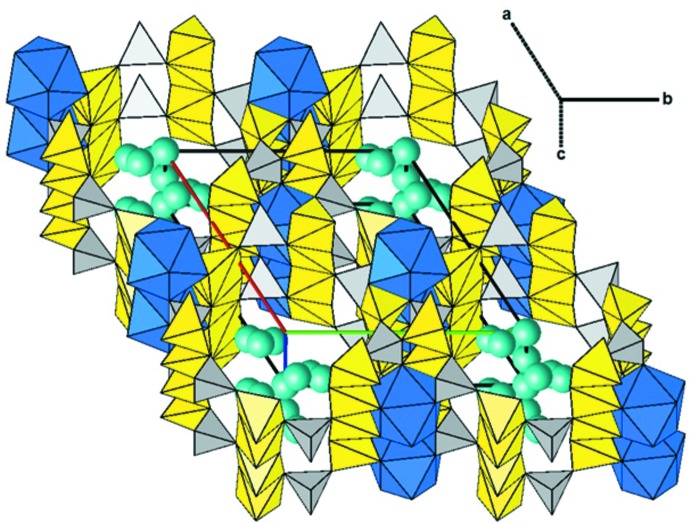
The crystal structure of agardite-(Y). Yellow square-pyramids, gray tetrahedra and blue polyhedra represent CuO5, AsO4 and YO9 units, respectively. Cyan spheres, with arbitrary radius, represent the O atoms of lattice water molecules.

**Fig. 3.**
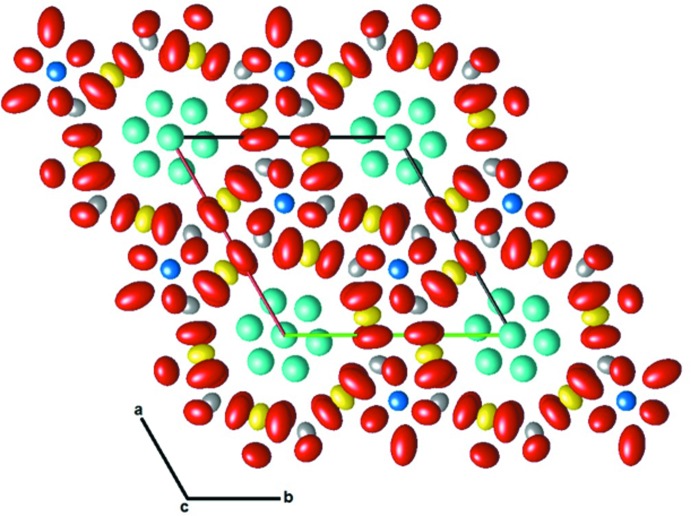
The crystal structure of agardite-(Y) represented with displacement ellipsoids at the 99% probability level. Yellow, gray, blue and red ellipsoids represent Cu, As, Y and O, respectively. O atoms of lattice water molecules, shown as cyan spheres, are represented with an arbitrary radius.

**Fig. 4.**
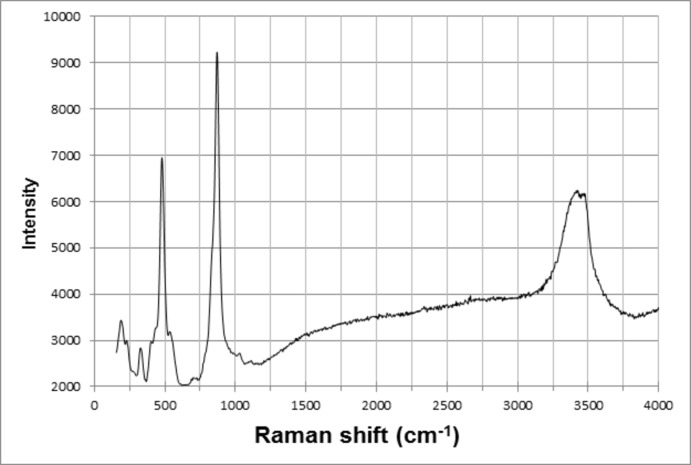
Raman spectrum of agardite-(Y). The broad water vibration band is centered at 3400 cm-1, and the two small bands protruding from the water band signifying two OH modes.

## supplementary crystallographic information

### 1. Comment

Minerals of the mixite group crystallize in the mixite-structure type in space group *P*6_3_/*m* and with *Z* = 2. Minerals of this and other groups, where the crystal structures exhibit channels (occupied by lattice water molecules), are of particular industrial and environmental interest due to their potential applications in the sorption of toxic chemicals (Leone *et al.*, 2013) and as catalysts (Miletich *et al.*, 1997; Frost *et al.*, 2005). The mixite group can be characterized by the general formula Cu^2+^_6_*A*(*T*O_4_)_3_(OH)_6_.3H_2_O, where the nine-coordinated *A* site represents a rare earth element (*REE*), Al, Ca, Pb, or Bi, and the *T* site is P or As. There are currently ten members of this group: mixite [Cu^2+^_6_Bi(AsO_4_)_3_(OH)_6_.3H_2_O], zálesíite [Cu_6_Ca(AsO_4_)_2_(AsO_3_OH)(OH)_6_.3H_2_O], agardite-(Ce) [Cu^2+^_6_Ce(AsO_4_)_3_(OH)_6_.3H_2_O], agardite-(La) [Cu^2+^_6_La(AsO_4_)_3_(OH)_6_.3H_2_O], agardite-(Nd) [Cu^2+^_6_Nd(AsO_4_)_3_(OH)_6_.3H_2_O], agardite-(Y) [Cu^2+^_6_Y(AsO_4_)_3_(OH)_6_.3H_2_O], goudeyite [Cu_6_Al(AsO_4_)_3_(OH)_6_.3H_2_O], plumboagardite [Cu_6_(Pb,La,Nd,Ce,Ca)(AsO_4_)_3_(OH)_6_.3H_2_O], petersite-(Y) [Cu_6_Y(PO_4_)_3_(OH)_6_.3H_2_O], and calciopetersite [Cu_6_Ca(PO_4_)_2_(PO_3_OH)(OH)_6_.3H_2_O].

Due to its small crystal size and acicular habit, only the crystal structures of agardite-(Ce) (Hess, 1983), mixite (Mereiter & Preisinger, 1986; Miletich *et al.*, 1997) and zálesíite (Aruga & Nakai, 1985) have been reported thus far. Notably, Aruga & Nakai (1985) studied a sample with composition [(Ca_0.40_*REE*_0.42_Fe_0.09_)Cu_6.19_[(AsO_4_)_2.42_(HAsO_4_)_0.49_](OH)_6.38_.3H_2_O] that they called a Ca-rich agardite. With the description of a new Ca-rich member of the mixite group, calciopetersite (Sejkora *et al.*, 2005), the sample studied by Aruga and Nakai (1985) should be called zálesíite (Hatert & Burke, 2008).

Agardite-(Y) was first described from the oxidation zone of the Bou-Skour copper deposit in Jebel Sahro, Morocco (Dietrich *et al.*, 1969). It has since been found in many other localities, including Germany, England, Spain, France, U.S. (Dietrich *et al.*, 1969), Italy (Olmi *et al.*, 1988), Czech Republic (Plášil *et al.*, 2009) and Bulgaria (Kunov *et al.*, 2002). In these studies, unit-cell parameters were presented, but no details of the crystal structure. Amid identification of minerals for the RRUFF project (http://rruff.info/R070649), we detected sprays of relatively large, well-crystalized, acicular agardite-(Y) from the Jote West mine, Pampa Larga Mining District, Copiapo, Chile (Fig. 1). Thereby, this study represents the first crystal structure determination of agardite-(Y), by means of single-crystal X-ray diffraction.

The structure of agardite-(Y) consists of infinite chains of edge-sharing CuO_5_ square-pyramids (site symmetry 1) extending down the *c*-axis, connected in the *ab*-plane by edge-sharing, YO_9_-polyhedra (site symmetry 6..) and corner-sharing AsO_4_-tetrahedra (site symmetry *m*..) (Fig. 2). Hydroxyl groups (OH4 & OH5) occupy each corner of the CuO_5_-polyhedra not shared by a neighboring As or Y atom. Based on bond valance calculations, OH4 (bond valance sum = 1.07 valence units (v.u.)) donates a hydrogen bond to O1 (bond valance sum = 1.93 v.u.), at the apex of the CuO_5_-polyhedron, while also accepting a hydrogen bond from OH5 (bond valance sum = 1.26 v.u.). Each YO_9_-polyhedron is surrounded by three tubular channels (Fig. 3). The walls of the channels, parallel to the *c*-axis, are 6-membered, hexagonal rings comprised of CuO_5_- and AsO_4_-polyhedra in a ratio of 2:1, respectively, and contain free molecules of lattice water. The water positions form a ring inside the channel, similar to the 2.7 Å radius ring reported by Hess (1983) in agardite-(Ce) and the five water sites reported by Miletich *et al.* (1997). In our model of agardite-(Y), we defined two distinct water sites, OW1 and OW2, although there are many statistically possible locations. OW1 is positioned as a 2.93 Å radius ring inside the channel and OW2 is situated at the center of the channel. This sample's Raman spectrum (Fig. 4) shows a broad H_2_O band, centered at 3400 cm^-1^, and protruding from it are two small bands signifying two OH modes.

Previous studies have utilized thermogravimetric analysis to examine the nature of both the lattice water and the Hydroxyl groups in synthetic mixite-group minerals (Miletich *et al.*, 1997; Frost *et al.*, 2005). In both studies, ~3 lattice (channel) H_2_O molecules were driven off when samples were heated to 373 K, and dehydroxylation was observed when temperatures reached 523 K. Mixite structural decomposition occurs upon the loss of the hydroxyl groups, which is made evident by the inability to rehydrate samples heated above the 523 K level (Miletich *et al.*, 1997). Previously, the lattice water was thought to also contribute to the stability of the mixite-group crystal structure; however, Miletich *et al.* (1997) showed a very low value of activation energy for dehydration in mixite, indicating that the water molecules are not bonded to any cation. Our findings support the hypothesis that such water molecules are not involved in bonding; Cu—OW bond lengths are >3.5 Å and bond valance calculations show Cu (valence sum = 2.11 v.u.) and As (valence sum = 5.19 v.u.) to be fully bonded. Therefore, it appears that lattice water is not essential to the stability of the agardite-(Y) crystal structure.

### 2. Experimental

The agardite-(Y) specimen used in this study was from the Jote West mine, Pampa Larga Mining District, Copiapo, Chile and is in the collection of the RRUFF project (deposition No. R070649; http://rruff.info). The chemical composition was determined with a CAMECA SX100 electron microprobe at the conditions of 25keV, 20nA, and a focused beam. An average of 16 analysis points yielded (wt. %): Al_2_O_3_ 0.59, P_2_O_5_ 3.09, CaO 3.72, MnO 0.10, CuO 43.18, As_2_O_5_ 30.92, Y_2_O_3_ 5.97, Gd_2_O_3_ 0.18, Tb_2_O_3_ 0.02, Dy_2_O_3_ 0.25, Ho_2_O_3_ 0.10, Er_2_O_3_ 0.32, Yb_2_O_3_ 0.22, H_2_O (by difference) 11.00. The empirical chemical formula is (Cu_5.33_Al_0.12_Ca_0.03_Mn_0.01_)_Σ__=5.49_(Y_0.52_Er_0.02_Dy_0.01_Yb_0.01_Ho_0.01_Ca_0.43_)_Σ__=1.00_[(AsO_4_)_0.88_(PO_4_)_0.14_]_3_(OH)_6_·2.99H_2_O.

### 3. Refinement

Due to similar X-ray scattering power, all *REE* were treated as Y. Y and Ca were allowed to share the *A*-site and their abundances were refined under consideration of full occupancy. Additionally, As and P were allowed to share the *T*-site and their abundances were also constrained under consideration of full occupancy. The occupancy of the Cu site was refined freely, revealing a slight underoccupation of 0.950 (9). Various models were attempted in refining the lattice water positions, including split-site models. However, the refinement adopted here is the only one that converged. The exceptionally large isotropic displacement parameters for OW1 and OW2 are expected because these site represent essentially free molecules in a large channel. The total number of O atoms for the two water sites was constrained to 6. H atoms could not be assigned reliably and were excluded from refinement. The highest residual peak in the difference Fourier maps was located at (0, 0, 0.5), 0.00 Å from OW2, and the deepest hole at (0.4927, 0.8006, 0.3668), 0.91 Å from As.

### Crystal data

**Table:**

Cu_5.70_(Y_0.69_Ca_0.31_)[(As_0.83_P_0.17_)O_4_]_3_(OH)_6_·3H_2_O	*D*_x_ = 3.519 Mg m^−^^3^
*M**_r_* = 985.85	Mo *K*α radiation, λ = 0.71073 Å
Hexagonal, *P*6_3_/*m*	Cell parameters from 786 reflections
Hall symbol: -P 6c	θ = 2.3–27.6°
*a* = 13.5059 (5) Å	µ = 13.13 mm^−^^1^
*c* = 5.8903 (2) Å	*T* = 293 K
*V* = 930.50 (6) Å^3^	Acicular needle, green
*Z* = 2	0.10 × 0.02 × 0.02 mm
*F*(000) = 936	

### Data collection

**Table:**

Bruker APEXII CCD diffractometer	786 independent reflections
Radiation source: fine-focus sealed tube	674 reflections with *I* > 2σ(*I*)
Graphite monochromator	*R*_int_ = 0.048
φ and ω scan	θ_max_ = 27.6°, θ_min_ = 3.0°
Absorption correction: multi-scan (*SADABS*; Bruker, 2004)	*h* = −17→17
*T*_min_ = 0.353, *T*_max_ = 0.779	*k* = −17→17
20461 measured reflections	*l* = −6→7

### Refinement

**Table:**

Refinement on *F*^2^	Primary atom site location: structure-invariant direct methods
Least-squares matrix: full	Secondary atom site location: difference Fourier map
*R*[*F*^2^ > 2σ(*F*^2^)] = 0.032	H-atom parameters not refined
*wR*(*F*^2^) = 0.086	*w* = 1/[σ^2^(*F*_o_^2^) + (0.0413*P*)^2^ + 5.6747*P*] where *P* = (*F*_o_^2^ + 2*F*_c_^2^)/3
*S* = 1.14	(Δ/σ)_max_ = 0.020
786 reflections	Δρ_max_ = 2.34 e Å^−^^3^
60 parameters	Δρ_min_ = −0.79 e Å^−^^3^
1 restraint	

### Special details

**Table:**

Geometry. All e.s.d.'s (except the e.s.d. in the dihedral angle between two l.s. planes) are estimated using the full covariance matrix. The cell e.s.d.'s are taken into account individually in the estimation of e.s.d.'s in distances, angles and torsion angles; correlations between e.s.d.'s in cell parameters are only used when they are defined by crystal symmetry. An approximate (isotropic) treatment of cell e.s.d.'s is used for estimating e.s.d.'s involving l.s. planes.
Refinement. Refinement of *F*^2^ against ALL reflections. The weighted *R*-factor *wR* and goodness of fit *S* are based on *F*^2^, conventional *R*-factors *R* are based on *F*, with *F* set to zero for negative *F*^2^. The threshold expression of *F*^2^ > σ(*F*^2^) is used only for calculating *R*-factors(gt) *etc*. and is not relevant to the choice of reflections for refinement. *R*-factors based on *F*^2^ are statistically about twice as large as those based on *F*, and *R*- factors based on ALL data will be even larger.

### Fractional atomic coordinates and isotropic or equivalent isotropic displacement parameters (Å^2^)

**Table:**

	*x*	*y*	*z*	*U*_iso_*/*U*_eq_	Occ. (<1)
Y	0.6667	0.3333	0.2500	0.0084 (5)	0.69 (2)
Ca	0.6667	0.3333	0.2500	0.0084 (5)	0.31 (2)
Cu	0.41303 (5)	0.31598 (5)	0.50234 (10)	0.0113 (2)	0.950 (9)
As	0.49505 (6)	0.15100 (6)	0.7500	0.0087 (3)	0.828 (15)
P	0.49505 (6)	0.15100 (6)	0.7500	0.0087 (3)	0.172 (15)
O1	0.5739 (3)	0.1820 (3)	0.5182 (6)	0.0161 (9)	
O2	0.3919 (5)	0.4007 (4)	0.2500	0.0180 (12)	
O3	0.4124 (5)	0.2123 (5)	0.7500	0.0180 (12)	
OH4	0.3688 (5)	0.3768 (5)	0.7500	0.0174 (12)	
OH5	0.4421 (5)	0.2456 (5)	0.2500	0.0233 (14)	
OW1	0.134 (3)	0.170 (3)	0.2500	0.221 (15)*	0.7676 (7)
OW2	0.0000	0.0000	0.5000	1.4 (4)*	0.697 (2)

### Atomic displacement parameters (Å^2^)

**Table:**

	*U*^11^	*U*^22^	*U*^33^	*U*^12^	*U*^13^	*U*^23^
Y	0.0102 (5)	0.0102 (5)	0.0048 (7)	0.0051 (3)	0.000	0.000
Ca	0.0102 (5)	0.0102 (5)	0.0048 (7)	0.0051 (3)	0.000	0.000
Cu	0.0183 (4)	0.0153 (4)	0.0042 (4)	0.0113 (3)	0.0006 (2)	0.0003 (2)
As	0.0116 (4)	0.0086 (4)	0.0055 (4)	0.0048 (3)	0.000	0.000
P	0.0116 (4)	0.0086 (4)	0.0055 (4)	0.0048 (3)	0.000	0.000
O1	0.0184 (18)	0.0214 (19)	0.0109 (17)	0.0118 (16)	0.0039 (14)	0.0024 (14)
O2	0.029 (3)	0.025 (3)	0.009 (2)	0.020 (3)	0.000	0.000
O3	0.025 (3)	0.020 (3)	0.012 (3)	0.013 (2)	0.000	0.000
OH4	0.027 (3)	0.023 (3)	0.009 (2)	0.017 (2)	0.000	0.000
OH5	0.042 (4)	0.026 (3)	0.011 (3)	0.023 (3)	0.000	0.000

### Geometric parameters (Å, º)

**Table:**

Y—O1^i^	2.384 (3)	Cu—OH5	1.908 (3)
Y—O1^ii^	2.384 (3)	Cu—OH4	1.911 (3)
Y—O1^iii^	2.384 (3)	Cu—O2	1.982 (3)
Y—O1	2.384 (3)	Cu—O3	2.019 (4)
Y—O1^iv^	2.384 (3)	Cu—O1^iv^	2.290 (4)
Y—O1^v^	2.384 (3)	As—O1^vi^	1.652 (3)
Y—OH5^ii^	2.647 (6)	As—O1	1.652 (3)
Y—OH5^iv^	2.647 (6)	As—O3	1.690 (5)
Y—OH5	2.647 (6)	As—O2^vii^	1.692 (5)
			
O1^i^—Y—O1^ii^	136.02 (6)	O1^v^—Y—OH5^iv^	67.84 (12)
O1^i^—Y—O1^iii^	80.86 (13)	OH5^ii^—Y—OH5^iv^	120.000 (1)
O1^ii^—Y—O1^iii^	83.01 (17)	O1^i^—Y—OH5	67.84 (12)
O1^i^—Y—O1	83.01 (17)	O1^ii^—Y—OH5	138.49 (8)
O1^ii^—Y—O1	80.86 (13)	O1^iii^—Y—OH5	138.49 (8)
O1^iii^—Y—O1	136.02 (6)	O1—Y—OH5	67.84 (12)
O1^i^—Y—O1^iv^	136.02 (6)	O1^iv^—Y—OH5	68.19 (12)
O1^ii^—Y—O1^iv^	80.86 (13)	O1^v^—Y—OH5	68.19 (12)
O1^iii^—Y—O1^iv^	136.02 (6)	OH5^ii^—Y—OH5	120.0
O1—Y—O1^iv^	80.86 (13)	OH5^iv^—Y—OH5	120.0
O1^i^—Y—O1^v^	80.86 (13)	OH5—Cu—OH4	174.6 (3)
O1^ii^—Y—O1^v^	136.02 (6)	OH5—Cu—O2	80.14 (17)
O1^iii^—Y—O1^v^	80.86 (13)	OH4—Cu—O2	99.09 (17)
O1—Y—O1^v^	136.02 (6)	OH5—Cu—O3	98.49 (17)
O1^iv^—Y—O1^v^	83.01 (17)	OH4—Cu—O3	81.53 (16)
O1^i^—Y—OH5^ii^	68.19 (12)	O2—Cu—O3	172.1 (2)
O1^ii^—Y—OH5^ii^	67.84 (12)	OH5—Cu—O1^iv^	84.2 (2)
O1^iii^—Y—OH5^ii^	67.84 (12)	OH4—Cu—O1^iv^	101.26 (19)
O1—Y—OH5^ii^	68.19 (12)	O2—Cu—O1^iv^	96.13 (18)
O1^iv^—Y—OH5^ii^	138.49 (8)	O3—Cu—O1^iv^	91.46 (18)
O1^v^—Y—OH5^ii^	138.49 (8)	O1^vi^—As—O1	111.5 (3)
O1^i^—Y—OH5^iv^	138.49 (8)	O1^vi^—As—O3	112.08 (15)
O1^ii^—Y—OH5^iv^	68.19 (12)	O1—As—O3	112.08 (15)
O1^iii^—Y—OH5^iv^	68.19 (12)	O1^vi^—As—O2^vii^	108.17 (16)
O1—Y—OH5^iv^	138.49 (8)	O1—As—O2^vii^	108.17 (16)
O1^iv^—Y—OH5^iv^	67.84 (12)	O3—As—O2^vii^	104.4 (3)

Symmetry codes: (i) *x*, *y*, −*z*+1/2; (ii) −*y*+1, *x*−*y*, *z*; (iii) −*y*+1, *x*−*y*, −*z*+1/2; (iv) −*x*+*y*+1, −*x*+1, *z*; (v) −*x*+*y*+1, −*x*+1, −*z*+1/2; (vi) *x*, *y*, −*z*+3/2; (vii) *y*, −*x*+*y*, −*z*+1.
